# Characteristics of neonates with culture-proven bloodstream infection who have low levels of C-reactive protein (≦10 mg/L)

**DOI:** 10.1186/s12879-015-1069-7

**Published:** 2015-08-11

**Authors:** Mei-Yin Lai, Ming-Horng Tsai, Chiang-Wen Lee, Ming-Chou Chiang, Reyin Lien, Ren-Huei Fu, Hsuan-Rong Huang, Shih-Ming Chu, Jen-Fu Hsu

**Affiliations:** Division of Pediatric Neonatology, Department of Pediatrics, Chang Gung Memorial Hospital, Taoyuan, Taiwan; Division of Neonatology and Pediatric Hematology/Oncology, Department of Pediatrics, Chang Gung Memorial Hospital, Yunlin, Taiwan; College of Medicine, Chang Gung University, Taoyuan, Taiwan; Department of Nursing, Division of Basic Medical Sciences, and Chronic Diseases and Health Promotion Research Center, Chang Gung University of Science and Technology, Chia-Yi, Taiwan; Research Center for Industry of Human Ecology, Chang Gung University of Science and Technology, Taoyuan, Taiwan; Linkou Chang Gung Memorial Hospital, No. 5, Fu-Shin Rd., Kwei-Shan, Taoyuan, Taiwan

## Abstract

**Background:**

Elevated C-reactive protein (CRP) level is widely used in clinical practice as a marker to distinguish between neonates with or without sepsis. However, some neonates with bacteremia have a CRP level within the normal range and they are not well characterized.

**Methods:**

All episodes of neonatal culture-proven bloodstream infections (BSIs) between July 2004 and June 2012 were enrolled. Patients characteristics were compared for three CRP groups (low, ≤ 10 mg/L; intermediate, 11–100 mg/L; and high, > 100 mg/L) using the Chi-square test and one-way ANOVA. The sepsis-attributable mortality rates were compared using logistic regression analyses.

**Results:**

Of 986 episodes of neonatal BSI, 247 (25.1 %) had CRP ≤10 mg/L at the onset of clinical sepsis. In the low CRP group, patients had lower gestational age and birth weight, and an earlier occurrence of BSI. Patients with underlying gastrointestinal pathology, renal disorders, cholestasis, and pulmonary hypertension had a non-significant elevated CRP level at the onset of sepsis. In the blood culture of the low CRP group, coagulase-negative staphylococci (CoNS) were relatively more common (55.9 %, *p* < 0.001) than the other two groups, although one-fourth were infected with gram-negative bacilli (19.0 %), fungi (2.8 %), or polymicrobial pathogens (3.6 %). Of the BSIs with initial low CRP, 29.1 % were treated with inadequate antibiotics, 13.0 % progressed to septic shock, and 5.3 % had infectious complications. The sepsis-attributable mortality rate was lower in the low CRP group (4.9 %) than in the high CRP group (13.6 %).

**Conclusions:**

A considerable proportion of neonatal BSIs had a normal or low initial CRP level (≤10 mg/L), which was more likely to occur in low birth weight or extremely preterm infants, those with earlier onset of sepsis, and those infected with CoNS. Plasma CRP level should not be used to rule out severe culture-proven sepsis or guide the empirical choice of antibiotics.

## Background

The incidence of culture-proven bloodstream infection (BSI) in the neonatal intensive care unit (NICU) is around 37 episodes per 10,000 neonate-hospital days [1–4]. Neonatal BSI is associated with significant morbidity, worse long-term outcomes, and a reported mortality rate of 12–20 % [1, 5–8]. Early and appropriate antibiotic treatment is the cornerstone of treatment [9, 10], which highlights the importance of a high index of suspicion based on clinical and laboratory parameters long before the blood culture results are known. However, some neonatal BSIs may present with nonspecific symptoms and several laboratory predictors have been investigated for better identification of bacteremia in the NICU [11–15].

C-reactive protein (CRP) is an acute-phase protein of hepatic origin that increases following interleukin-6 secretion from macrophages and T-cells [16, 17]. The synthesis of CRP in response to inflammation starts very rapidly, and serum concentration rises to > 5 mg/L after 6 h and peaks at 48 h [18, 19]. The CRP reference values in neonates reported in the literature are 1.5–20 mg/L and have a wide range of sensitivities and specificities [19, 20]. Although some studies have demonstrated that at least 2 CRP levels, both ≤ 10 mg/L and obtained 24 h apart, are needed to identify infants unlikely to be infected [19, 21], the high cut-off value associated with bacterial infection in neonates is still uncertain. Conversely, a small proportion of patients with bacteremia have CRP levels within the normal range [22, 23]. To date, this issue has not been well studied in the NICU. The present study was therefore conducted to characterize outcomes in neonates with culture-proven BSI and a CRP plasma level ≤ 10 mg/L.

## Methods

We identified all episodes of culture-proven BSI in the NICU of Chang Gung Memorial Hospital (CGMH), a tertiary-level medical center in a university-affiliated teaching hospital in northern Taiwan, between 1 July 2004 and 30 June 2012. Only infants with late-onset sepsis, defined as clinical sepsis with a positive blood culture obtained after 3 days of life [1, 3, 24], were enrolled. The NICU has 3 units that include 49 beds equipped with mechanical ventilators and 28 beds in special-care nurseries. All babies under 34–35 weeks of completed gestation or birth weight < 2 kg or > 5 kg or those with any clinical signs of respiratory distress or cardiovascular, gastrointestinal, or neurological problems requiring surgical or intensive treatment were eligible for admission to our NICU. This study was approved by the institutional review board of Chang Gung Memorial Hospital, with a waiver of informed consent because all patient records/information was anonymized and de-identified prior to analysis.

### Data retrieval and study database

Information for the NICU database was recorded by a full-time nurse specialist dedicated to following up neonates from birth (or admission if the neonate was transferred from another hospital) until discharge or death, and this nurse specialist has maintained the database every weekday for > 10 y. The NICU database contained information about basic demographic data, a summary of the patient’s hospital course, discharge diagnosis, and complications during prematurity. Medical records were reviewed to characterize the detailed courses of every episode of BSI, including the clinical manifestations, laboratory results, progression of septic conditions, concurrent infectious focus, treatment, infectious complications, and outcomes. The severity of illness was evaluated at the most severe period during the course of BSI using the neonatal therapeutic intervention scoring system (NTISS) [25].

### Definitions

An episode of BSI was defined according to the presence of clinical sepsis and identification of pathogens, which included any bacteria isolated from at least one blood culture and not pertaining to the saprophytic skin flora as significant. Growth of *Corynebacterium* spp., *Propionibacterium* spp., *Penicillium* spp., or *Diphtheroids* spp. in the blood cultures were considered contaminants and were excluded. The microbiology assay system in our hospital is a matrix-assisted laser desorption ionization time-of-flight (MALDI-TOF) system (Bruker’s flagship FLEX series). For coagulase-negative staphylococci (CoNS) BSI, the diagnosis required clinical signs of sepsis, a blood culture positive for CoNS, and administration of intravenous antibacterial therapy for at least 5 days after the blood culture or until death [1, 2, 7].

An episode of clinical sepsis was defined if a patient had a positive blood culture treated with antibiotic therapy for 5 or more days or treated for a shorter period if the patient died, and the presence of at least two of the following clinical symptoms of sepsis: fever or hypothermia, hyper- or hypoglycemia, apnea or tachypnea, frequent desaturation with an increased requirement for ventilator support, bradycardia and/or cyanosis, feeding intolerance, abdominal distension, seizure, decreased activity, skin mottling and hypotension [1, 7, 9].

All co-morbidities of prematurity, including respiratory distress syndrome (RDS), intra-ventricular hemorrhage (IVH), broncho-pulmonary dysplasia (BPD), necrotizing enterocolitis (NEC), and peri-ventricular leukomalacia (PVL) were based on the latest updated diagnostic criteria [26]. Congenital anomalies included all neonates with either documented or undocumented syndrome, chromosome abnormalities, and genetic or metabolic disorders, but not simple cleft palate or polydactyly. Persistent bacteremia or fungemia was defined as three or more consecutive positive blood cultures, at least 48 h apart, during a single episode of sepsis [27]. All concurrent infectious foci, including NEC, ventilator associated pneumonia (VAP), catheter-related bloodstream infection (CRBSI), or meningitis were also recorded based on the strict diagnostic criteria of the Centers for Disease Control and Prevention and previous official publications [28, 29].

Infectious complications were defined as a new-onset infectious focus, empyema, abscess, venous thrombosis, or vegetation directly related to bacteremia or major organ dysfunction within 1 week after the onset of bacteremia. Empirical antibiotic therapy was considered inappropriate if the treatment regimen did not include at least 1 antibiotic active in vitro against the infecting microorganisms administered within 24 h of blood culture collection. For patients who died during hospitalization, the cause of death was recorded according to the clinician’s assessment. Sepsis-attributable mortality was defined as neonates who expired within 3 days after the onset of sepsis and those who died of infectious complications or clinically progressive deterioration following the onset of BSI.

### Data on CRP

The CRP plasma level was measured with an immunoturbidimetric instrument (Modular P, Roche, Germany). We excluded episodes of BSI for which CRP was not measured on the date of BSI onset. If more than 1 CRP measurement was done on the date of BSI, we used the measurement conducted closest to the time when blood culture samples were drawn. In the laboratory, a CRP level ≤ 5 mg/L was considered within the normal range. The low limit of detection of CRP in our hospital is > 0.5 mg/L, and there is no upper limit of CRP detection. All BSI episodes were divided into 3 CRP groups: low (CRP ≤ 10 mg/L), intermediate (CRP 11–100 mg/L), and high (CRP > 100 mg/L).

### Statistical analysis

The patient characteristics by CRP group were computed in contingency tables. Differences in categorical and continuous variables were tested by the χ^2^ test and one-way ANOVA, respectively. For continuous variables with large standard deviations, the nonparametric Kruskal–Wallis *H* test was applied. Trends in proportions of microorganisms in the 3 CRP groups were analyzed using the χ^2^ test for trend. Statistical significance was set at *P* < 0.05.

The sepsis-attributable mortality rate by CRP group was examined using a logistic regression model. Univariate and multivariate analyses, including the co-variables of sex, gestational age, underlying chronic co-morbidities, and bacterial subgroups, were performed. Because of the strong correlation between birth weight and gestational age, the risk factor of body weight at birth was excluded from the analysis. Estimates were expressed as odds ratios (ORs) with 95 % confidence intervals (CIs). All statistical analyses were performed using SPSS version 15.0 (SPSS, Chicago, IL, USA).

## Results

During the study period, 1010 episodes of BSI (late-onset sepsis) in 793 neonates from the NICU were identified. Those with incomplete medical records (*n* = 8), no CRP measurement at BSI onset (*n* = 12), or transfer to another hospital (*n* = 4) were excluded, leaving a total of 986 BSI episodes in 772 neonates. Among these, 247 (25.1 %) had a plasma CRP level ≤ 10 mg/L, 563 (57.1 %) had a plasma CRP level of 11–100 mg/L, and 176 (17.8 %) had a plasma CRP level > 100 mg/L.

Patients in the low CRP group had a lower birth body weight and gestational age and an earlier onset of BSI than patients in the intermediate and high CRP groups (Table 1). There were no significant differences in the perinatal history and most underlying chronic co-morbidities among the 3 CRP groups. However, patients with gastrointestinal pathology, pulmonary hypertension, renal disorders, or cholestasis were more likely to have high CRP at BSI onset. When the clinical symptoms or signs were compared, significantly more patients in the high CRP group had apnea, bradycardia with or without cyanosis, feeding intolerance, hyper- or hypoglycemia, or septic shock compared to those in the low CRP group (Table 1). These clinical symptoms were not significantly different between the low and intermediate CRP groups.

**Table 1:** Episodes of bloodstream infection stratified according to low (CRP 0–10 mg/L), intermediate (CRP 11–100 mg/L) and high (CRP > 100 mg/L) level

Characteristics	CRP 0–10 mg/L (*n* = 247)	CRP 11–100 mg/L (*n* = 563)	CRP > 100 mg/L (*n* = 176)	*p* value
Demographic characteristics				
Birth weight (g), mean ± SD	1310 ± 746	1507.9 ± 809	1725.2 ± 959	0.004
median (IQR)	1310 (920–1740)	1530 (885–1975)	1685 (923–2610)	
Gestational age (weeks), mean ± SD	30.2 ± 4.3	30.5 ± 4.6	31.4 ± 5.2	0.036
median (IQR)	30.0 (27.0–33.0)	30.0 (27.0–34.0)	30.0 (27.0–36.0)	
Male sex, n (%)	140 (56.7)	292 (51.9)	88 (50.0)	0.154
Onset of BSI (day), median (IQR)	23.0 (15.0–43.0)	30.0 (18.0–56.0)	32.5 (19.0–63.8)	<0.001
Perinatal history				
NSD/CS	112 (45.3)/135 (54.7)	220 (39.1)/343 (60.9)	81 (46.0)/95 (54.0)	0.901
Inborn/outborn	163 (66.0)/84 (34.0)	381 (67.7)/182 (32.3)	112 (63.6)/64 (36.4)	0.697
Low apgar score at 5 min (≦7)	111 (44.9)	255 (45.3)	70 (39.8)	0.344
Perinatal asphyxia	18 (7.3)	48 (8.5)	11 (6.3)	0.738
Underlying chronic conditions^a^				
Congenital anomalies	15 (6.1)	35 (6.2)	18 (10.2)	0.128
Neurological comorbidities	35 (14.2)	63 (11.2)	31 (17.6)	0.443
Complicated congenital heart disease (CHD)	8 (3.2)	23 (4.1)	4 (2.3)	0.696
Acyanotic CHD with heart failure	5 (2.0)	9 (1.6)	7 (4.0)	0.234
Bronchopulmonary dysplasia	66 (26.7)	188 (33.4)	60 (34.1)	0.087
Pulmonary hypertension	3 (1.2)	18 (3.2)	11 (6.3)	0.004
Gastrointestinal pathology	4 (1.6)	47 (8.3)	15 (8.5)	0.002
Renal	6 (2.4)	17 (3.0)	12 (6.8)	0.024
Cholestasis^b^	30 (12.1)	117 (20.8)	41 (23.3)	0.002
Clinical septic symptoms				
Fever (temperature > 38 °C)	99 (40.1)	228 (40.5)	84 (47.7)	0.135
Apnea ± bradycardia and/or cyanosis	158 (64.0)	385 (68.4)	139 (79.0)	0.001
Abdominal distension ± feeding intolerance	130 (52.6)	328 (58.3)	139 (79.0)	<0.001
Tachycardia	55 (22.3)	134 (23.8)	35 (19.9)	0.652
Hyper- or hypoglycemia	53 (21.5)	157 (27.9)	55 (31.3)	0.020
Septic shock	32 (13.0)	93 (16.5)	45 (25.6)	0.001
Disseminated intravascular Coagulopathy	12 (4.9)	45 (8.0)	30 (17.0)	<0.001
Laboratory parameter				
Leukopenia (WBC count < 4000/uL)	25 (10.1)	109 (19.4)	38 (21.6)	0.001
Leukocytosis (WBC count > 20,000/uL)	62 (25.1)	151 (26.8)	64 (36.4)	0.017
WBC shift to left (immature WBC ≥ 20 % total)	13 (5.3)	65 (11.5)	44 (25.0)	<0.001
Anemia (hemoglobin < 11.0 mg/dL)	79 (32.5)	217 (38.5)	98 (55.7)	<0.001
Thrombocytopenia (platelet < 80,000/uL)	41 (16.6)	197 (35.0)	110 (62.5)	<0.001
Metabolic acidosis	26 (10.7)	104 (18.5)	60 (34.1)	<0.001
Prolonged PT and/or aPTT	34 (14.0)	150 (26.6)	70 (39.8)	<0.001
NTISS score, mean ± SD	16.3 ± 4.2	16.9 ± 4.6	18.2 ± 5.1	<0.001
Sequences of BSI during hospitalization				0.014
First episode	204 (82.6)	412 (73.2)	128 (72.7)	
2nd episode	29 (11.7)	95 (16.9)	31 (17.6)	
>2nd episode	14 (5.7)	56 (9.9)	17 (9.7)	
Concomitant infectious focus^c^				
Meningitis	12 (4.9)	20 (3.6)	23 (13.1)	0.001
Catheter-related bloodstream infection	12 (4.9)	30 (5.3)	20 (11.4)	0.012
Necrotizing enterocolitis (≥ stage II)^d^	4 (1.6)	10 (1.8)	7 (4.0)	0.126
Ventilator associated pneumonia	7 (2.8)	18 (3.2)	15 (8.5)	0.007
Others^e^	1 (0.4)	3 (0.5)	3 (1.7)	-

All data were expressed as number (percentage %), unless indicated otherwise

*NTISS* neonatal therapeutic intervention scoring system, *IQR* interquartile range, *SD* standard deviation, *BSI* bloodstream infection, *NSD* natural vaginal delivery, *C/S* cesarean section

^a^Indicating the presence of chronic conditions or comorbidities at onset of bloodstream infection, and some patients had > 1 underlying chronic conditions. Each patient with an underlying chronic condition is compared with those without that specific condition

^b^Indicating direct bilirubin ≥ 1.5 mg/dL or more than 50 % of the total bilirubin

^c^Indicating the diagnosis of a infectious focus was concurrent with onset of bacteremia

^d^Based on modified Bell’s staging criteria

^e^Included osteomyelitis (2), septic arthritis (2), left thigh cellulitis (1), and urinary tract infection (2)

Episodes of BSI in the high CRP group had a significantly higher rate of abnormal laboratory findings, including leucopenia, anemia, thrombocytopenia, and metabolic acidosis compared to those in the low CRP group. The severity of illness, judged by NTISS score, was also significantly higher in the high CRP group than in the low CRP group. These demographics, clinical features, and laboratory findings were not significantly different between neonatal BSIs in the low and intermediate CRP groups as determined by post hoc analyses (data not shown). However, neonatal BSIs in the low CRP groups occurred significantly earlier than in both the intermediate and high CRP groups. Episodes of BSI with concurrent meningitis, ventilator-associated pneumonia, and catheter-related BSI also had elevated CRP at the onset of clinical sepsis (Table 1).

### Microbiology

In the low CRP group, gram-positive, gram-negative, fungal, and polymicrobial pathogens accounted for 74.5, 19.0, 2.8, and 3.6 % of infections, respectively. CoNS was significantly more common in the blood cultures of the low CRP group compared to the other groups. A lower proportion of most gram-negative bacteria occurred in the low CRP group (Table 2). However, for gram-positive bacteria, there were significantly fewer patients with Group B *Streptococcus* BSI and comparable numbers of *Staphylococcus aureus* BSI in the low CRP group.

**Table 2:** Pathogens causing neonatal bloodstream infections in the three CRP groups

Pathogens	CRP 0–10 mg/L (*n* = 247)	CRP 11–100 mg/L (*n* = 563)	CRP > 100 mg/L (*n* = 176)	*P* value
Gram-positive organisms	184 (74.5)	322 (57.2)	63 (35.8)	<0.001
Coagulase-negative staphylococci (CoNS)	138 (55.9)	227 (40.3)	24 (13.6)	<0.001
*Staphylococcus aureus*	34 (13.8)	62 (11.0)	23 (13.1)	0.715
*Enterococcus* spp.	8 (3.2)	18 (3.2)	1 (0.6)	0.130
*Group-B streptococcus*	3 (1.2)	13 (2.3)	15 (8.5)	<0.001
Others^a^	1 (0.4)	2 (0.4)	0 (0)	-
Gram-negative organisms	47 (19.0)	174 (30.9)	91 (51.7)	<0.001
*E coli*	12 (4.9)	41 (7.3)	23 (13.1)	0.003
*Klebsiella* spp.	18 (7.3)	66 (11.7)	32 (18.2)	0.001
*Enterobacter* spp.	4 (1.6)	26 (4.6)	11 (6.3)	0.015
*Acinetobacter baumannii*	6 (2.4)	23 (4.1)	12 (6.8)	0.028
*Pseudomonas* spp.	3 (1.2)	6 (1.1)	6 (3.4)	0.103
*Serratia marscences*	3 (1.2)	7 (1.2)	1 (0.6)	0.574
Others^b^	2 (0.8)	5 (0.9)	6 (3.4)	0.113
Fungemia	7 (2.8)	38 (6.7)	11 (6.3)	0.090
Polymicrobial bloodstream infection	9 (3.6)	29 (5.2)	11 (6.3)	0.214

^a^Including *Streptococcus pneumonia* (1), and non-enterococcal group D streptococcus (2)

^b^Including *Citrobacter freundii* (3), *Stenotrophomonas maltophilia* (3), *Hafnia alvei* (2), Neisseria Meningitidis (2), *Chryseobacterium meningoseptium* (1) *Flavobacterium* (1) and *Morganella morganii* (1)

Of the 247 patients with an initial plasma CRP level ≤ 10 mg/L, 178 had at least 1 more CRP measurement during the days following BSI onset. Among these, 82 (46.1 %) developed a CRP response > 10 mg/L (median [IQR]: 35.0 mg/L [22.8–52.5 mg/L]) at a median of 2.8 days (range 1–7 days) after antibiotic treatment. Of the 138 episodes of CoNS BSI with an initial CRP plasma level ≤ 10 mg/L, 58 (42.0 %) were treated with initial antibiotics that were inadequate and had repeated positive blood cultures. Among the 96 episodes of neonatal BSIs with repeated CRP data that still had a CRP level ≤ 10 mg/L, 31 (32.3 %) had CoNS in their blood cultures.

### Treatments and outcomes

**Fig. 1:**
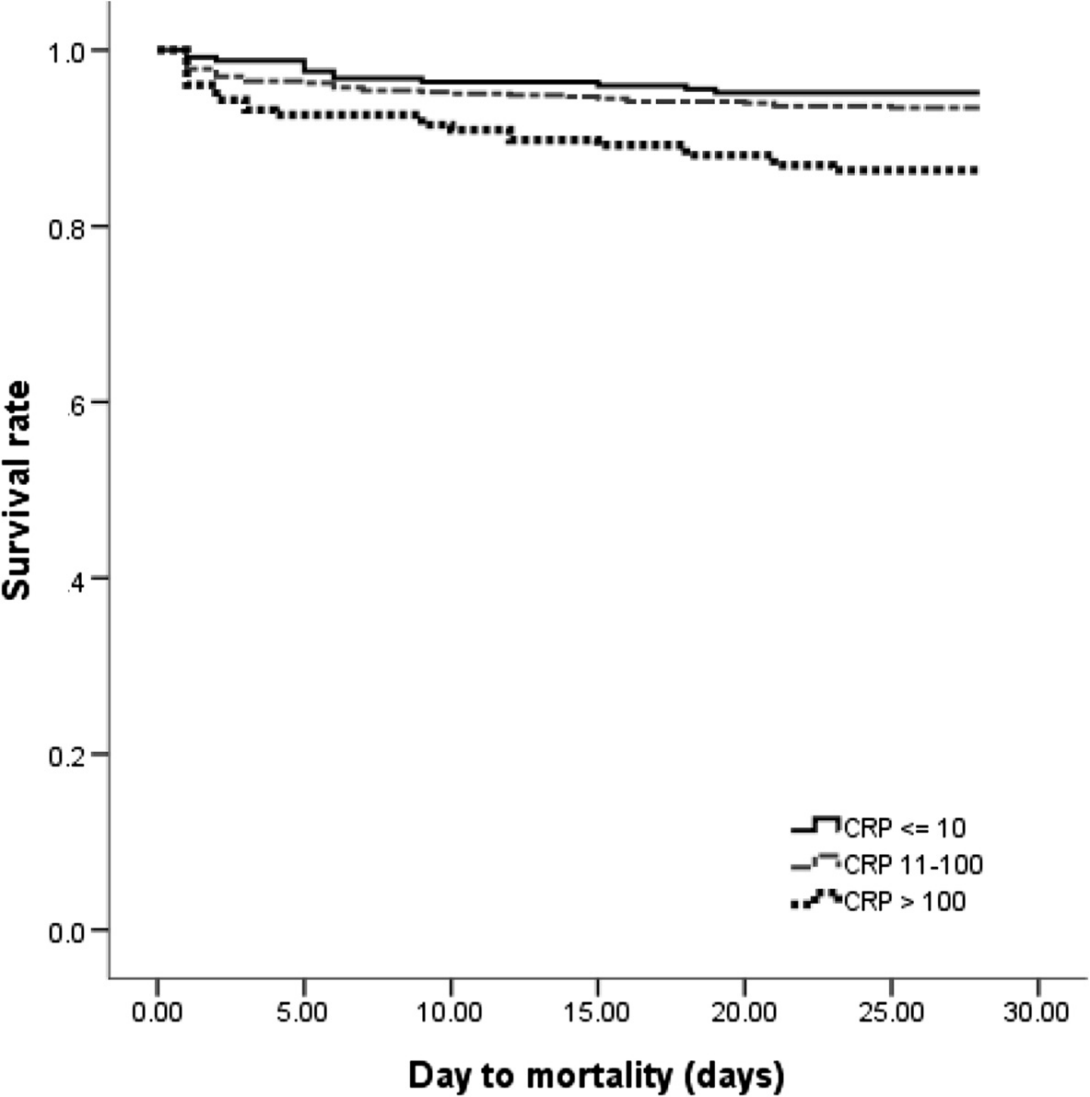
Survival following positive blood culture from 986 episodes of neonatal culture-proven bloodstream infection. The Kaplan-Meier graph is stratified by three different CRP groups: low, ≤ 10 mg/L; intermediate, 11–100 mg/L; and high, > 100 mg/L

Neonates who experienced BSI episodes and had higher CRP levels had significantly more invasive intubation or high-frequency oscillatory ventilator use (Table 3). The rates of inadequate antibiotic treatment were comparable among the 3 CRP groups. The overall sepsis-attributable mortality rate was 7.4 % and occurred in 4.9, 6.6, and 13.6 % in the low, intermediate, and high CRP groups, respectively. Neonates in the high CRP group had a significantly higher sepsis-attributable mortality compared with those in the intermediate and low CRP groups (*P* < 0.05 by log-rank test) (Fig. 1).

**Table 3:** Treatment and outcomes of neonatal bloodstream infections among the three CRP groups

	CRP 0–10 mg/L (*n* = 247)	CRP 11–100 mg/L (*n* = 563)	CRP > 100 mg/L (*n* = 176)	*P* value
Treatment				
Invasive intubation	106 (42.9)	254 (45.1)	95 (54.0)	0.042
High frequency oscillatory ventilator	18 (7.3)	57 (10.1)	25 (14.2)	0.021
Inadequate antibiotic treatment with the first 24 h	72 (29.1)	150 (26.6)	42 (23.9)	0.463
Outcomes				
Infectious complications	13 (5.3)	49 (8.7)	42 (23.9)	<0.001
Sepsis attributable mortality	12 (4.9)	37 (6.6)	24 (13.6)	0.001

There was no difference in the sepsis-attributable mortality rate between those with CRP ≤ 10 mg/L and those with CRP of 11–100 mg/L. The CRP level was a predictor of sepsis-attributable mortality (OR 3.09, 95 % CI 1.50–6.37) in the high CRP group compared to the low CRP group. The difference between these 2 groups remained unchanged even after adjustments for birth weight, sex, gestational age, bacterial subgroups, and several underlying chronic morbidities (data not shown).

## Discussion

Approximately one-fourth of the cases of neonatal BSI—which is a surprisingly high proportion in the current cohort—had a CRP level of ≤ 10 mg/L at the onset of clinical sepsis. Low birth weight neonates, very low gestational age infants, and those in whom the occurrence of BSI episodes occurred earlier in life were more likely to have normal CRP at the onset of clinical sepsis. We found a high CRP level (>100 mg/L) was significantly associated with more severe clinical symptoms and worse outcomes. Although CoNS accounts for more than half of the episodes of BSI with normal CRP, in our cohort approximately one-fourth of those with normal CRP were infected with gram-negative bacteria, polymicrobial pathogens, or fungus. Furthermore, approximately 30 % of those with normal CRP were treated with inadequate initial antibiotics, and 13 % progressed to septic shock and 4.9 % had sepsis-attributable mortality.

It is reasonable to hypothesize that causative pathogens affect CRP level because some pathogens, especially gram-negative bacilli or *Pseudomonas* spp., tend to have a fulminant course [1, 7, 9], which can result in more severe tissue damage and inflammation. The findings here are consistent with a recent study conducted by Knudtzen et al., who found that CoNS BSI is more common in the low CRP group and that approximately 25–35 % of all CoNS BSI have normal CRP at the onset of clinical sepsis [22]. Because drawing 2 sets of blood culture at the same time is not routinely done and the second blood culture may become negative if vancomycin was initially administrated, the lack of 2 positive blood cultures for documentation of CoNS BSI is a limitation of this study. Some possibly represent bacterial contaminations. To reduce the false positive cases of CoNS BSI, strict criteria for CoNS BSI have been applied, including requiring more than 2 septic symptoms or signs, as in previous studies [1, 2, 8]. Besides, more than three-fourths of the CoNS BSIs have elevated CRP or abnormal laboratory findings.

Because the initial symptoms of neonatal sepsis may be nonspecific and subtle and may become fulminant if appropriate antibiotics are not administered in time, the role of plasma CRP level in adequately predicting serious bacterial infection in neonates has recently become the topic of several studies [30–33]. Although we found that significantly more infectious complications and sepsis-attributable mortality occurred in the CRP group with > 100 mg/L, a possibly delayed elevation and inadequate sensitivity of CRP testing highlight the importance of investigating other predictors, including new laboratory markers [30–35] and clinical scores [36–38] and maintaining high alertness for the development of severe sepsis. Several studies report a sensitivity and specificity of 48–78 % and 71–88 %, respectively, for a single determination of CRP at 4–12 h after symptom onset [31–35]. Therefore, taken together, using the CRP level as a guide for empirical choice of antibiotics or to rule out serious infections or withhold antibiotic therapy is not recommended.

This study also investigated whether BSI episodes with initially low CRP levels developed a CRP response later. In contrast to a recent study in which 84 % of BSIs with initially normal CRP mounted a CRP response [22], the current study reveals that only 46 % have elevated subsequent CRP. This may be due to the high rate of initial effective antibiotic therapy and, possibly, the presence of CoNS as a low-level pathogenic bacterium that causes less tissue damage and inflammation. In a cohort of 60 neonates with early-onset sepsis, Ehl et al. [39] demonstrated that the CRP values decrease 16 h after successful antibiotic therapy. Some cases without CRP response in our cohort presented with a very fulminant course and hepatic failure, which was similar to a finding in a previous study [40].

The value of CRP as a predictive marker for bacterial infection in neonates has been studied with varying results [30–34, 38]. However, most of these studies focused on serious bacterial sepsis or critically ill neonates. This study aimed to characterize the NICU patients with normal or low CRP levels at the onset of sepsis and compared them with other neonates who had an elevated CRP level. We found the differences were most significant between neonatal BSIs in the low CRP group and those in the high CRP (>100 mg/L) group. The non-significant difference between the low CRP and the intermediate CRP groups by post hoc analyses (data not shown) implied that initial CRP level cannot be the sole predictor of final mortality. In this study, patients in the high CRP group treated with inappropriate antibiotics in the first 24 h were at especially higher risk of infectious complications and sepsis attributable mortality. However, those in the low CRP group had a relatively smooth clinical course even though they were initially treated with delayed effective antibiotics. Therefore, CRP still has a reference value as an indicator of severe infection.

We found that patients with lower gestational age or lower birth body weight tended to have a low CRP value at the onset of BSI. Because CRP is the end product of cytokine response after tissue injury, it is likely that extremely preterm or very low birth weight infants have less pronounced immunomodulatory or pre-inflammatory proteins after bacterial infection [41, 42]. Of interest, we found this cytokine response less severe in earlier life, but further investigation to validate these differences is required because the CRP level can also be affected by various factors, including pathogens, severity of illness, concomitant infectious focus, and some underlying chronic comorbidities.

There are some limitations in this study. Although a set of strict criteria was used to define true BSI, the lack of 2 positive blood cultures for CoNS as documentation of BSI is a weakness of this study. This was a retrospective, single-center cohort study, which inevitably restricts its generalizability compared to that of a prospective, multicenter study. The timing of blood sampling for culture and CRP also was not uniform because some septic work-up may be delayed because the initial septic symptoms were nonspecific and subtle. Furthermore, some CRP levels may have been falsely low because they were obtained too early before they started to rise around 4–6 h after sepsis onset. Lastly, a group with elevated CRP but without infection was not enrolled as controls. Thus, a prospective study is warranted to scientifically delineate the sensitivity and specificity of CRP as a predictive marker of neonatal sepsis.

## Conclusion

Nearly one-forth of neonatal bloodstream infections had a normal or low initial CRP level (<=10 mg/L), which was more likely to occur in low birth weight or extremely preterm infants, those with earlier onset of sepsis, and those infected with coagulase-negative Staphylococcus. Although initial low CRP level was significantly with less severity of illness and better outcome, some critically ill neonates had CRP not significantly elevated at onset of sepsis. Therefore, plasma CRP level should not be used to rule out severe culture-proven sepsis or guide the empirical choice of antibiotics.

## Abbreviations

BSI: Bloodstream infection
CDC: Center for disease control
BPD: Bronchopulmonary dysplasia
CI: Confidence interval
CRBSI: Catheter-related bloodstream infection
CoNS: Coagulase negative Staphylococcus
CRP: C-reactive protein
CVC: Central venous catheter
ESBL: Extended-spectrum β-lactamase
GBS: Group B streptococcus
IQR: Interquartile range
GPC: Gram-positive cocci
MALDI-TOF: Matrix-assisted laser desorption ionization time-of-flight
NEC: Necrotizing enterocolitis
NICU: Neonatal intensive care unit
NTISS: Neonatal therapeutic intervention scoring system
OR: Odds ratio
PVL: Periventricular leukomalacia
RDS: Respiratory distress syndrome
TPN: Total parenteral nutrition
VAP: Ventilator associated pneumonia (VAP)
